# COVID-19 Vaccine Hesitancy Associated With Vaccine Inequity Among Healthcare Workers in a Low-Income Fragile Nation

**DOI:** 10.3389/fpubh.2022.914943

**Published:** 2022-07-11

**Authors:** Mohammed Noushad, Mohammad Zakaria Nassani, Mohammed Sadeg Al-Awar, Inas Shakeeb Al-Saqqaf, Sami Osman Abuzied Mohammed, Abdulaziz Samran, Ali Ango Yaroko, Ali Barakat, Omar Salad Elmi, Anas B. Alsalhani, Yousef Fouad Talic, Samer Rastam

**Affiliations:** ^1^College of Dentistry, Dar Al Uloom University, Riyadh, Saudi Arabia; ^2^Faculty of Applied Science, Amran University, Amran, Yemen; ^3^Department of Medical Laboratory, College of Medical Science, Al-Razi University, Sana'a, Yemen; ^4^School of Social Sciences, University Sains Malaysia, George Town, Malaysia; ^5^Emergency Department, King Abdullah Medical City, Mecca, Saudi Arabia; ^6^Otolaryngology Department, Usman Danfodiyo University Teaching Hospital, Sokoto, Nigeria; ^7^School of Population and Global Health, University of Melbourne, Melbourne, VIC, Australia; ^8^Vision College of Dentistry and Nursing, Vision Colleges, Riyadh, Saudi Arabia; ^9^Vision College of Medicine, Vision Colleges, Riyadh, Saudi Arabia

**Keywords:** vaccine acceptance, low-income country, Yemen, lack of access, COVID-19

## Abstract

**Objectives:**

Preventing severe disease and acquiring population immunity to COVID-19 requires global immunization coverage through mass vaccination. While high-income countries are battling vaccine hesitancy, low-income and fragile nations are facing the double dilemma of vaccine hesitancy and lack of access to vaccines. There is inadequate information on any correlation between vaccine hesitancy and access to vaccines. Our study in a low-income nation aimed to fill this gap.

**Methods:**

In the backdrop of a severe shortage of COVID-19 vaccines in Yemen, a low-income fragile nation, we conducted a nation-wide cross-sectional survey among its healthcare workers (HCWs), between 6 July and 10 August 2021. We evaluated factors influencing agreement to accept a COVID-19 vaccine and any potential correlation between vaccine acceptance and lack of access to vaccines.

**Results:**

Overall, 61.7% (*n* = 975) of the 1,581 HCWs agreed to accept a COVID-19 vaccine. Only 45.4% of the participants agreed to have access to a COVID-19 vaccine, with no sex dependent variations. Although several determinants of vaccine acceptance were identified, including, having a systemic disease, following the updates about COVID-19 vaccines, complying with preventive guidelines, having greater anxiety about contracting COVID-19, previous infection with COVID-19, believing COVID-19 to be a severe disease, and lower concern about the side effects of COVID-19, the strongest was access to vaccines (OR: 3.18; 95% CI: 2.5–4.03; *p*-value: 0.001).

**Conclusion:**

The immediate and more dangerous threat in Yemen toward achieving population immunity is the severe shortage and lack of access to vaccines, rather than vaccine hesitancy, meaning, improving access to vaccines could lead to greater acceptance.

## Introduction

The Coronavirus disease 2019 (COVID-19) pandemic has been a global public health threat for more than 2 years, causing more than 516 million infections and 6.25 million deaths (1). There are also suggestions that globally, at least half of the COVID-19 deaths have been thought to be missed. For example, it has been suggested that the percentage of deaths from COVID-19 reported in the United States is just 78%, and in a highly populated country like India, it is only 10% indicating a public health burden greater than reported (2). This is especially true in resource-poor and conflict countries where intentional or unintentional largescale mortality underreporting, shortages in testing capacity and availability of health care workers (HCWs) are major concerns (3). So far, the World Health Organization (WHO) has identified more than thirteen variants of the SARS-CoV-2 that causes COVID-19, and it is expected to mutate further, unless global population immunity is successfully achieved (4). Currently, the favored pathway to reach that goal is through successful global vaccination programs.

Although several countries are still facing recurrent waves of the virus transmission, the implementation of robust vaccination programs have been instrumental in limiting morbidity and mortality from COVID-19. However, the optimum immunization coverage necessary to reach global population immunity can be achieved only by regulated and inclusive vaccine distribution covering all countries regardless of country income index.

Since healthcare workers (HCWs) work in the frontline in the fight against COVID-19, they are one of the most vulnerable. According to the World Health Organization (WHO), an estimated 80,000 to 180 000 health and care workers could have died from COVID-19 between January 2020 and May 2021 (5). Due to their vulnerability, and to prevent a potential breakdown of the healthcare system, globally they have been prioritized to receive vaccination against COVID-19. Thanks to the implementation of coordinated vaccination programs, as of September 2021, more than 80% of the HCWs in 22 mostly high-income countries (HIC) have been fully vaccinated against COVID-19 (5). Unfortunately, these figures are overshadowed by considerable differences across regions and economic groups. For example, as of November 2021, only 27% of HCWs in the African continent have been fully vaccinated against COVID-19, a result of extreme vaccine inequity (6).

While several HICs are battling vaccine hesitancy in pursuit to achieve maximum vaccine coverage, it is vaccine inequity that has crippled low-income countries (LIC). Even after a year since the approval of several vaccines against COVID-19, LICs are still struggling with extreme shortage in vaccine supply, enough to fully vaccinate only a fraction of their populations, while HICs have fully vaccinated more than 73% of their populations (7). Numerous studies have been conducted worldwide on vaccine hesitancy/acceptance among HCWs and the general population. However, there is inadequate information on the correlation between vaccine hesitancy/acceptance and access to vaccines. In the backdrop of a severe vaccine shortage in Yemen, we conducted an exploratory cross-sectional study among HCWs in Yemen, a low-income conflict nation, to identify predictors of COVID-19 vaccine acceptance and any potential correlation with lack of access to vaccines.

## Materials and Methods

### Study Design

This study is part of a large project on global COVID-19 vaccine acceptance. Therefore, the methodology and the questionnaire followed is similar across all the studies (8). A cross-sectional self-administered survey was conducted among HCWs in Yemen between 6 July and 10 August 2021. The “Report of the SAGE working group on vaccine hesitancy” was used as a guide in preparing the questionnaire (9). As part of the validation of the study, a pilot study was initially carried out on 10 participants, after which expert opinions were taken from specialists in the field. The survey questionnaire, developed on Google Forms, was distributed by dual mode (online and paper based) to prospective participants. The questionnaire required <5 min to complete. Participation was voluntary and the participants provided informed consent on the survey platform before proceeding to the survey items. Participants were not asked to disclose their names or email addresses, and their anonymity was guaranteed during the data collection process. The survey form was designed in such a way that only complete forms would qualify for submission.

This study was approved by the Research Committee of College of Dentistry, Dar Al Uloom University, Riyadh, Saudi Arabia (COD/IRB/2020/2).

### Sample

The sample size was calculated using Open Source Epidemiologic Statistics for Public Health–OpenEpi (http://www.openepi.com/Menu/OE_Menu.htm, accessed on 25 June 2021). We used 50% as the 'hypothesized percentage frequency of the outcome factor (vaccine acceptance) in the population, which is recommended for an unknown frequency, and 4% as the absolute precision. The resultant sample size for 99% confidence interval using these parameters was 1,036.

Participants were recruited using convenience sampling. Participants included HCWs from all governorates of Yemen. Participants below the age of 18 years were not included in the study. Participants were not paid compensation for participation in the study.

### Measures

#### Trust in COVID-19 Vaccines, Health Authorities and the International Community, and Access to Vaccines

General attitudes toward vaccines were measured using a 2-item scale and participants' attitudes toward the health authorities were measured using a 4-item scale. Participants were then asked if they had access to COVID-19 vaccines. Responses were rated on a five-point Likert scale from 1 “strongly agree” to 5 “strongly disagree” (8).

#### Intention to Vaccinate

This was measured using a 7-item scale. Responses were rated on a five-point Likert scale from 1 “strongly agree” to 5 “strongly disagree” (8).

### Predictor Variables

Socio-demographic factors included age group, sex, nationality, place of work and region. Participants' reports on chronic medical conditions (e.g., asthma, diabetes, hypertension, heart disease, and/or cancer) were used to indicate the presence or absence of pre-existing co-morbidity. Other variables included participants' self-updating on COVID-19 vaccine development, prior infection with COVID-19, perception of COVID-19 severity, compliance with government COVID-19 guidelines, and anxiety toward contracting COVID-19.

### Statistical Analysis

Descriptive statistics were expressed as percentages and numbers for each item/survey question. The main outcome of this study was the agreement to accept a COVID-19 vaccine and potential correlation with access to vaccines. The current study considered any participant to have an intention to vaccinate if he/she agreed or strongly agreed on the item “I will get vaccinated with the COVID-19 vaccine,” or if they had already taken the vaccine. Bivariate statistical analysis of the relationship between the main outcome “agreement to accept a COVID-19 vaccine” and demographic and other parameters was performed using the Chi-squared test for trend for ordinal factors, and the Chi-squared test for categorical variables. A multivariate binary logistic regression model was used to determine the predictors for intention to vaccinate. The following factors were examined as potential predictors for “intention to vaccinate:” age group, sex, nationality, presence of any medical condition, following updates on the development of vaccines against COVID-19, opinion about the severity of COVID-19, compliance with COVID-19 preventive guidelines, and anxiety about contracting COVID-19, previous COVID-19 infection, concerns about side effects of COVID-19 vaccines and access to COVID-19 vaccines. We chose possible covariates based on biological plausibility, and all factors that showed significant results in the bivariate analysis. The significance level was set at *p* < 0.05. All statistical analyses were performed using IBM SPSS Statistics version 25.0 (IBM Corp. Released 2017. IBM SPSS Statistics for Windows, Version 25.0. Armonk, NY, USA: IBM Corp).

## Results

Overall, 1,581 HCWs completed the survey questionnaire (response rate 73%). Of the participants, 61.7% (*n* = 975) expressed their agreement to get vaccinated against COVID-19. Males (54.1%) and females (45.9%) were equally distributed. The majority of the participants were from the 19–29 years age group (62.4%) and from the Azal province (52.6%). The majority of the participants had no comorbidities (81.8%). Characteristics and demographics of the participants are shown in Table 1. Data regarding participants' awareness about COVID-19 are presented in Table 2. It can be noted that more than a quarter of the participants (27.6%) were previously infected with COVID-19, while only 10.9% got vaccinated against COVID-19. At least 74.3% of the participants agreed that COVID-19 is a threat to public health in Yemen, and 81.8% have been updating themselves on the development of COVID-19 vaccines. Less than a half of the participants (39%) rated COVID-19 as a severe disease. Just 7.1% expressed their poor compliance with COVID-19 preventive guidelines, and only 12.7% agreed that they were highly anxious about contracting COVID-19 (Table 2).

**Table 1:** Sample characteristics: data are presented as *n* (%).

**Sample size**	***n*** **(%)**
Total	1,581
**Sex**	
Male	855 (54.1%)
Female	726 (45.9%)
**Age**	
18–29 years	987 (62.4%)
30–49 years	387 (24.5%)
≥50 years	207 (13.1%)
**Nationality**	
Yemeni	1,580 (99.9%)
Foreigner	1 (0.1%)
**Province**	
Aden province	164 (10.4%)
Azal province	832 (52.6%)
Hadhramout province	101 (6.4%)
Jund province	168 (10.6%)
Sheba province	189 (12%)
Tihama province	127 (8%)
**Place of work**	
Public	487 (30.8%)
Private	911 (57.6%)
Both	183 (11.6%)
**Work**	
Doctor	142 (9%)
Lab specialist/medical technician	120 (7.6%)
Dentist	158 (10%)
Nurse/dental assistant/midwife	206 (13%)
Pharmacist	409 (25.9%)
Physiotherapist, epidemiology, nutrition	138 (8.7%)
Health care student	137 (8.7%)
Other	271 (17.1%)
**Comorbidity**	
No	1293 (81.8%)
Yes	288 (18.2%)

**Table 2:** Awareness about COVID-19 infection.

	***n*** **(%)**
**Have you been updating yourself on the development of vaccine?**	
No	288 (18.2%)
Yes	1,293 (81.8%)
**In your opinion, how would you rate the severity of COVID-19 disease:**	
Mild	132 (8.3%)
Moderate	833 (52.7%)
Severe	616 (39%)
**How would you rate your compliance with COVID-19 preventive guidelines?**	
Good	846 (53.5%)
Moderate	622 (39.3%)
Poor	113 (7.1%)
**To what extent are you anxious about contracting (getting infected with)**	
**COVID-19?**	
Low	524 (33.1%)
Moderate	856 (54.1%)
High	201 (12.7%)
**Have you had COVID-19?**	
No	1,144 (72.4%)
Yes	437 (27.6%)
**COVID-19 is a threat to public health in Yemen**	
Agree	1,175 (74.3%)
Not sure	261 (16.5%)
Disagree	145 (9.2%)
**Have you taken the COVID-19 vaccine?**	
No	1,408 (89.1%)
Yes	173 (10.9%)

*Data are presented as n (%)*.

67.4% of the participants agreed that vaccines against COVID-19 are important to overcome the pandemic, 69.1% were concerned about their side effects and 44.1% thought that the vaccines have been produced in a hurry without following guidelines. 69.8% of the participants agreed that it was important for them to get vaccinated in order to protect those with weaker immune systems, and almost half of them (44.8%) were prepared to delay getting vaccinated for those who deserved it more than them. 68.4% of the participants agreed to pay to get vaccinated. In terms of pandemic management and organization of vaccination campaigns by the health authorities, 45.9 and 42.8% expressed their satisfaction, respectively. Similarly, less than half the participants expressed their satisfaction with the support provided by non-governmental organizations (NGO) (48.6%) and the international community (48.1%). Support for a mandatory vaccination program against COVID-19 was expressed by just 41.5% of the participants. 45.4% of the participants agreed that they have access to a COVID-19 vaccine. The abovementioned results are summarized in Table 3.

**Table 3:** Trust in COVID-19 Vaccines and Health Authorities, and access to vaccines.

	**Yemen**
Vaccines are necessary to overcome the COVID-19 pandemic and get back to normal life	1,065 (67.4%)
I am concerned about the possible side effects of COVID-19 vaccines	1,092 (69.1%)
I will delay taking the COVID-19 vaccine, as I feel there are others who deserve it more than me	708 (44.8%)
Getting myself vaccinated for COVID-19 is important because I can also protect people with a weaker immune system	1,103 (69.8%)
I will take the COVID-19 vaccine only if it is free	500 (31.6%)
I think that vaccines against COVID-19 have been produced in a hurry without following recommended clinical trials and approval guidelines	698 (44.1%)
I am happy with the way the health authorities have been managing the COVID-19 pandemic so far	726 (45.9%)
I am happy with the health authorities' organization of the COVID-19 vaccination campaigns	677 (42.8%)
I am happy with the way the Non-governmental organizations like the World Health Organization, Medicines Sans Frontiers, etc., have been helping my country in vaccinating its population	768 (48.6%)
I am happy with the way the international community is helping my country in vaccinating its population	761 (48.1%)
I support a mandatory vaccination program for COVID-19	646 (41.5%)
I have access to the COVID-19 vaccine	711 (45.4%)

*Data are presented as n (%)*.

The bivariate statistical analysis indicated a possible association between participants' intention to vaccinate and eight factors (*p* < 0.05; Table 4). The intention to get vaccinated increased significantly with increase in age and in those with systemic disease/s. Updating self on the development of COVID-19 vaccines, increasing severity perception about COVID-19, increasing compliance with preventive guidelines, a higher level of anxiety about contracting COVID-19, and lack of concern about the side effects of COVID-19 vaccines were all associated with a greater agreement to get vaccinated. Importantly, access to COVID-19 vaccine was significantly associated with a higher intention to get vaccinated. Details of the above results are presented in Table 4. There was no gender-based association with access to vaccines (Supplementary Table).

**Table 4:** Bivariate statistical analysis of the relationship between the main outcome “intention to vaccinate” and potential influential factors.

	**Yemen**	***p***
All participants	61.7% (975/1,581)	
**Age**		0.006
18–29 years	59.3% (585/987)	
30–49 years	64.1% (248/387)	
50 years or above	68.6% (142/207)^*^	
**Gender**		0.66
Male	61.2% (523/855)	
Female	62.3% (452/726)	
**Medical condition**		0.001
Healthy	59.8% (773/1,293)	
Has systemic disease/s	70.1% (202/288)^**^	
**Updating self on the development of vaccines against COVID-19**		<0.001
No	43.8% (126/288)	
Yes	65.7% (849/1293)^**^	
**Opinion about the severity of COVID-19**		<0.001
Mild	42.4% (56/132)	
Moderate	59.8% (498/833)	
Severe	68.3% (421/616)^*^	
**Compliance with COVID-19 preventive guidelines**		<0.001
Poor	33.6% (38/113)	
Moderate	57.4% (357/622)	
Good	68.6% (580/846)^*^	
**Anxiety about contracting COVID-19**		<0.001
Low	46.9% (246/524)	
Moderate	65.5% (561/856)	
High	83.6% (168/201)^*^	
**Previously infected with COVID-19**		0.60
No	62.1% (710/1144)	
Yes	60.6% (265/437)	
**Concerned about the possible side effects of COVID-19 vaccines**		<0.001
No	72% (352/489)	
Yes	57.1% (623/1092)^**^	
**Access to the COVID-19 vaccine**		<0.001
No	48.2% (413/856)	
Yes	78.3% (557/711)^**^	

* *p was calculated using chi-square test for trend*.

** *p was calculated using chi-square test. Significance difference was set at p < 0.05*.

*The denominators indicate the total number of participants in this subgroup, and the numerators are the number of participants who agreed to accept a vaccine*.

The logistic regression analysis indicated a possible association between agreement to get vaccinated against COVID-19 and six factors. These include having a systemic disease (OR: 1.49 95% CI: 1.03–2.16; p-value: 0.03), following the updates about COVID-19 vaccines (OR: 1.92; 95% CI: 1.43–2.57; *p*-value: 0.001), those who believed COVID-19 to be a severe disease, those who complied with preventive guidelines, those with greater anxiety about contracting COVID-19 (OR: 3.31; 95% CI: 2.08–5.25; *p*-value: 0.001), previous infection with COVID-19, those less concerned about the side effects of COVID-19 vaccines (OR: 0.49; 95% CI: 0.38–0.64; *p*-value: 0.001) and those who have access to a COVID-19 vaccine (OR: 3.18; 95% CI: 2.5–4.03; *p*-value: 0.001; Table 5). Of these, compliance with preventive guidelines, anxiety about contracting COVID-19, and access to a COVID-19 vaccine were showed to have the greatest association.

**Table 5:** Predictors of intention to vaccinate.

	**Odds ratio (95% CI)**	***p***
**Age**		
18–29 years	Ref	
30–49 years	1.37 (1.03–1.81)^*^	0.03
50 years or above	1.02 (0.66–1.56)	0.94
**Gender**		
Male	Ref	
Female	0.99 (0.79–1.25)	0.94
**Medical condition**		
Healthy	Ref	
Has systemic disease/s	1.49 (1.03–2.16)^*^	0.03
**Updating self on the development of vaccines against COVID-19**		
No	Ref	
Yes	1.92 (1.43–2.57)^*^	<0.001
**Opinion about the severity of COVID-19**		
Mild	Ref	
Moderate	1.61 (1.05–2.48)^*^	0.03
Severe	1.85 (1.18–2.91)^*^	0.007
**Compliance with COVID-19 preventive guidelines**		
Poor	Ref	
Moderate	2.1 (1.29–3.42)^*^	<0.001
Good	3.36 (2.07–5.43)^*^	0.003
**Anxiety about contracting COVID-19**		
Low	Ref	
Moderate	1.4 (1.08–1.8)^*^	0.01
High	3.31 (2.08–5.25)^*^	<0.001
**Previously infected with COVID-19**		
No	Ref	
Yes	0.85 (0.65–1.1)	<0.001
**Concerned about the possible side effects of COVID-19 vaccines**		
No	Ref	
Yes	0.49 (0.38–0.64)^*^	<0.001
**Access to the COVID-19 vaccine**		
No	Ref	
Yes	3.18 (2.5–4.03)^*^	<0.001

*Odds ratio and 95% confidence interval was calculated by a binary logistic model*.

* *Significant difference at p < 0.05*.

## Discussion

The success in the fight against COVID-19 rests largely on optimum immunization coverage through equitable vaccine distribution. Our study aimed to identify potential determinants of COVID-19 vaccine acceptance and any possible correlation with lack of access to vaccines, in Yemen, a low-income fragile nation. Although we did a similar study on the general population in Yemen, the current study looked for any similarities or differences with the previous study (10). Interestingly, the overall vaccine acceptance rate indicated by participants in our study (61.7%) is comparable to that indicated by HCWs in a similar study we conducted in neighboring Saudi Arabia (64.1%), a high-income country (11). It is however greater than that indicated by the general population, in the similar study we conducted in Yemen (10). Our results are comparable to other studies in HICs in the region, including one on more than 15,000 HCWs in Saudi Arabia (64.9%), one on HCWs in the United Arab Emirates (57.6%) and another one in the general population in Oman (56.8%) (12–14).

Studies in other fragile nations have indicated wide ranging results. For example, in an early multi-country study in Palestine, Syria and Jordan, it was shown that only 32.2% of the participants intended to be vaccinated against COVID-19 (15). Similarly, in two studies in Syria, the agreement to be vaccinated against COVID-19 was indicated by only about 37% of the participants (16, 17). A study in Iraq among HCWs and the general population revealed a vaccine acceptance rate similar (61.7%) to that of our study, with HCWs indicating higher acceptance rate than the general population (18). However, a study in Somalia indicated a higher acceptance rate of 76.8%, with HCWs indicating greater willingness to accept a COVID-19 vaccine than the general population (19). These results should encourage policymakers to leverage HCWs as a means of vaccine promotion among the general public since they have been shown to be trusted sources of information on vaccines (19, 20).

As in most countries, the burden of COVID-19 in Yemen is still unclear, the main reason being a severe shortage in testing capacity and availability of HCWs (3). Although a geospatial grave counting study in Yemen could not attribute all of the excess deaths to COVID-19, a seroprevalence study conducted in Aden in November 2020 indicates that the virus transmission is far higher than reported (21, 22). Nonetheless, the agreement of at least 74% of the participants on COVID-19 being a public health threat in Yemen, and almost 82% updating themselves on the development of vaccines against COVID-19 indicate their high level of awareness on COVID-19 and the vaccines against it. The challenge now lies in supplying enough doses to fill the gap between supply and demand.

Apart from demographic and other factors (Table 5), the strongest determinant of vaccine acceptance in our study was access to vaccines, which is similar to our study on the general population in Yemen (10). Even though access to COVID-19 vaccines was slightly higher among HCWs than the general population (39.9%), only 45.4% of the participants in the current study definitely agreed that they have access to a COVID-19 vaccine, and a mere 10.9% indicated having taken the COVID-19 vaccine (10). This is in sharp contrast to a recent study on vaccine intention among HCWs in neighboring Saudi Arabia, in which all the participants reported having been vaccinated against COVID-19 (23). Fragile nations like Yemen are faced with the double dilemma of vaccine shortage and logistic concerns related to the conflict. For example, in a study in Libya, 71.6% of the participants believed that COVID-19 vaccine distribution would be difficult, given the conflict related challenges there (24). Although access to vaccination facilities can be inconvenient to women and children, especially in low-income and fragile nations, as indicated in our study on the general population in Yemen, this variation may not be apparent among HCWs. With vaccine access rates of 45.7% in males and 45% in females, there was no sex-based differences in access to vaccines among participants in our current study. Unlike the general public, since participants in our study are HCWs who have been prioritized to receive vaccination against COVID-19, this finding is logical.

As of 13 March 2022, just 1.2% of the Yemeni population of more than 30 million have been fully vaccinated against COVID-19, while those in HICs is more than 73%. Moreover, during the same period, while the number of COVID-19 vaccine doses administered per 100 people in HICs is 192.17, that in LICs is a mere 19.52 (7). Although Yemen was promised a supply of 1.9 million vaccine doses throughout 2021, so far it has not received even a third of that number (25). These huge discrepancies and broken promises could lead to further decay of trust in policymakers and the international community. Our results indicate a low level of trust among HCWs in Yemen on NGOs (48.6%) and the international community (48.1%). This should prompt policymakers and stakeholders to take immediate action to gain back the trust, which could indirectly lead to greater vaccine acceptance.

Our results suggest that the immediate and greater threat in Yemen toward achieving population immunity is the lack of access to vaccines, rather than vaccine hesitancy. This is in agreement with a previous similar study on the general population in Yemen, highlighting the importance of provision of access to vaccines in low-income and fragile nations (10). Moreover, apart from vaccine inequity, due to the conflict related conditions, residents of these countries, especially women and children, are faced with the additional challenge of difficulty in accessing vaccination facilities. At a time when HICs are racing to provide booster/third dose of the vaccine, consideration should be given to simultaneously accelerate vaccine supplies in low-income and fragile nations, to achieve the minimum threshold necessary to attain population immunity. In light of the emergence of new variants of the wild-type virus, achieving population immunity in LICs is not only critical to protecting their populations, but it is also in the best interest of attaining global population immunity. The WHO, other NGOs operating in Yemen and other low-income and fragile nations, the COVAX and donor nations should work determinedly and inclusively with all parties and stakeholders to ensure that no one is left behind in the pursuit to achieve optimum vaccine coverage.

Notable strengths of our study include the wide coverage of the respondents spanning over all the provinces of Yemen, representing different demographic characteristics, and the large sample size. Moreover, this is the first study on vaccine acceptance among HCWs in Yemen following vaccine rollout, and the first study to assess any potential correlation between vaccine acceptance and lack of access to vaccines among HCWs. A limitation of our study is the inclusion of only complete questionnaires, which could affect the response rate, and the high number of participants from a particular age group (18–29 years old). Another limitation of our study is the web-based administration of the survey questionnaire. Since participants in our study included only those with access to internet facilities, there is a possibility for bias. Moreover, the web-based administration could be a cause for lower motivation of the participants to complete the questionnaire. However, due to the conflict related conditions in Yemen and the COVID-19 related restrictions, this was the best mode currently available. Since the rate of vaccine acceptance of the population could change over time, especially as more vaccines become available and accessibility increases, further studies will provide added value on the evolving vaccine acceptance trend among the public in Yemen. Similar studies in other low-income and fragile nations will provide a wider and global perspective on the correlation between vaccine acceptance and access to vaccines.

## Conclusions

Our results in Yemen, a low-income conflict country suggests vaccine acceptance comparable to those of neighboring countries. The potential correlation between vaccine acceptance and access to vaccines however indicates that a potential increase in supply will lead to an increase in demand. This should prompt policymakers to regulate vaccine supply to ensure that sufficient vaccines are distributed to low-income countries as well. Our results also send a strong message to policymakers on the importance of provision of access to vaccines to LICs and fragile nations in the event of possible future pandemics as well.

## Data Availability Statement

The raw data supporting the conclusions of this article will be made available by the authors, without undue reservation.

## Ethics Statement

This study was approved by the Research Committee of College of Dentistry, Dar Al Uloom University, Riyadh, Saudi Arabia (COD/IRB/2020/2). The patients/participants provided their written informed consent to participate in this study.

## Author Contributions

MN and MZN: conceptualization, methodology, and writing—original draft. MN, MZN, MA-A, IA-S, SM, AS, AY, AB, OE, AA, YT, and SR: investigation, data collection, and writing—review for important intellectual content and editing. SR, MN, and MZN: data analysis. All authors approved the final draft of the manuscript.

## Conflict of Interest

The authors declare that the research was conducted in the absence of any commercial or financial relationships that could be construed as a potential conflict of interest.

## Publisher's Note

All claims expressed in this article are solely those of the authors and do not necessarily represent those of their affiliated organizations, or those of the publisher, the editors and the reviewers. Any product that may be evaluated in this article, or claim that may be made by its manufacturer, is not guaranteed or endorsed by the publisher.
