# Supplementary material for: COVID-19 Vaccine Hesitancy Associated With Vaccine Inequity Among Healthcare Workers in a Low-Income Fragile Nation
Source: Front Public Health. 2022 Jul 11;10:914943. doi: 10.3389/fpubh.2022.914943 (PMC9309570; doi:10.3389/fpubh.2022.914943)
Supplement: Supplementary file 1 [file Table_1.pdf]

Supplementary table: Bivariate statistical analysis of the relationship between the "access to vaccine" and main demographics and health condition

	%	p
**All participants**	45.4% (711 / 1581)	-
**Age**		
18-29 years	45.5% (446 / 981)	0.25*
30-49 years	40.7% (155 / 381)	
50 years or above	53.7% (110 / 205)	
**Gender**		
Male	45.7% (387 / 847)	0.78**
Female	45% (324 / 720)	
**Place of work**		
Public	49.6% (240 / 484)	0.018**
Private	42.3% (383 / 905)	
Both	49.4% (88 / 178)	
**Medical condition**		
Healthy	44.7% (572 / 1281)	0.23**
Has systemic disease/s	48.6% (139 / 286)	

*: p was calculated using chi-square test for trend. **: p was calculated using chi-square test. Significance difference was set at p<0.05.
